# Food Insecurity and Coping Mechanisms: Impact on Maternal Mental Health and Child Malnutrition †

**DOI:** 10.3390/nu17020330

**Published:** 2025-01-17

**Authors:** Berna Rahi, Farah Al Mashharawi, Hana Harb, Myriam El Khoury-Malhame, Lama Mattar

**Affiliations:** 1 Department of Human Sciences, College of Health Sciences, Sam Houston State University, Huntsville, TX 77341, USA; bxa051@shsu.edu; 2 Nutrition Program, Department of Nutrition and Food Science, School of Arts and Sciences, Lebanese American University, Chouran Beirut, P.O. Box 13-5053, Beirut 1102 2801, Lebanon; farah.almashharawi@lau.edu (F.A.M.); hana.harb@lau.edu (H.H.); 3 Psychology Program, Department of Social Sciences, School of Arts and Sciences, Lebanese American University, Chouran Beirut, P.O. Box 13-5053, Beirut 1102 2801, Lebanon; myriam.malhame@lau.edu.lb

**Keywords:** food insecurity, mental health, coping mechanisms, maternal depression, child malnutrition

## Abstract

Background: Household food insecurity (HFI) is a serious public health concern in Lebanon. Adverse mental health issues have been reported among food insecure households in addition to physical and nutritional complications. Caregivers in food insecure families tend to adopt different coping mechanisms to mitigate the effects of food insecurity (FI) on their children. Objective: This cross-sectional observational study aimed to explore the relationship between FI, maternal depression, child malnutrition, and differential coping mechanisms adopted by mothers. Methods: A total of 219 women were enrolled in this study; FI was assessed using the household food insecurity assessment (HFIAS), maternal depression using the patient health questionnaire (PHQ-9), and their children’s nutritional status through recall of anthropometric measurements. Pearson’s correlations and logistic regressions were performed to evaluate the associations between HFI, maternal depression, and children’s nutritional status. Results: A strong positive correlation between HFI and maternal depression (*p* = 0.001) and children’s nutritional status (*p* = 0.008) was shown. Logistic regressions revealed that being food secure decreased the risk of maternal depression (OR = 0.328, 95% CI 0.125–0.863, *p* = 0.024), while it did not predict children’s nutritional status. Eight main themes related to coping mechanisms were identified. Conclusions: This study highlights the understudied relationship between food insecurity and maternal depression, showing an increased prevalence of HFI among residents in Lebanon with a positive correlation with increased maternal depression. Further investigation is warranted to better explore how to mitigate the negative impact of food insecurity on mental health, maternal nutritional needs, and Infant and Young Child Feeding (IYCF) practices in Lebanon.

## 1. Introduction

Food insecurity (FI) is a rising global problem, referring to the lack of consistent access to sufficient food for a healthy life [1]. FI is linked to adverse nutritional, physical, and mental health outcomes [2,3,4]. These include, but are not limited to, a higher risk of disease, increased risk of developmental, cognitive, and behavioral problems, nutritional deficiencies, poor physical health, early child growth faltering, obesity, and lower psychological functioning [4,5,6]. Furthermore, FI families report higher levels of anxiety, depression, stress, substance use, and somatic symptom disorders [2,7]. Not knowing when the next meal is, uncertainty over the ability to keep food supplies, and/or inability to acquire enough food in the future are main contributors to the stress response provoked by FI [2,7]. Additionally, not being able to obtain food in socially acceptable ways can be a key contributor to the feelings of guilt, shame, powerlessness and alienation [2,7,8,9]. Anxiety and depression are particularly high among FI women, considered as the primary caregivers of children in most households [10,11]. A recent cross-sectional study in fact examined the link between maternal mental health and high FI in Egypt and found that severely food insecure mothers were 13 times more likely to suffer from mental distress compared to their food secure peers [12]. Another consequence from FI is child malnutrition, which is considered to be one of the most severe consequences, especially in children under the age of 5 years [13].

Along with the adverse physical, mental, and nutritional outcomes, food insecure caregiver, especially mothers, usually adopt several coping mechanisms to attenuate the drawbacks of household food insecurity (HFI), [14,15]. The most common reported coping behaviors among HFI are purchasing food on credit and prioritizing children’s consumption over adults [16]. These reveal an underlying effect of gender-power relations on intra-household food distribution. A cross-sectional mixed methods comparative study in Nigeria found that maternal buffering was used as a preferential coping mechanism, meaning mothers skipped more meals or ate less food than other family members as a solution to HFI [17]. Other more drastic strategies are documented in another study in Yemen, indicating that some families resort to decreasing meal frequency altogether, borrowing food from others, changing the quality and portions of food, and even sending their children to relatives’ homes for survival and food provision [14]. In Lebanon, FI households cope by using leftovers from food waste [18,19]. As such, the dynamic interplay between FI, mental health, and coping mechanisms seems highly cultural and remains understudied.

### Current Context

Lebanon is a small Middle Eastern country enduring accumulating challenges, including a massive, unprecedented economic collapse, a banking crisis with a major devaluation of the local currency, decreased purchasing power, and high inflation rates. The country is also experiencing severe sociopolitical turmoil, reduced access to basic services, and the lasting effects of the Beirut port explosion and the COVID-19 pandemic, which have led to significant human and infrastructural losses [5]. Additionally, the country hosts the largest number of refugees per capita and per square kilometer, with approximately 1.5 million Syrian refugees and 13,715 refugees of other nationalities [20]. These crises have weakened the already fragile financial situation and subsequently threatened the physical and mental health of people residing in Lebanon and diminished their capacity to cope with distress [21]. It has also impelled a high level of FI and poverty [5,22]. The alarming continuous rise in FI in Lebanon [2,22,23] reflects a multifaceted situation encompassing factors such as the lack of affordability and accessibility of food, cultural standards influencing acceptable methods of obtaining food, and the personal utilization of food resources [6,7,24]. Recent data have indeed reported around one in four Lebanese households and four in ten Syrian refugees’ households residing in Lebanon faced daily shortages in food in 2020 [25]. The following year, a cross-sectional study revealed that 34.4% were moderately to severely food insecure, with 12.5% being severely food insecure [26]. The most recent report on the FI situation in Lebanon highlights that the situation is expected to worsen between April and September 2024; around 1.14 million people residing in Lebanon are expected to face high levels of FI (21% of the analyzed population). Specifically, a total of 582,000 Lebanese residents (15 percent of the resident population), 500,000 Syrian refugees (33 percent of the total Syrians in Lebanon), 49,000 PRL (27 percent of the Palestinian refugees in Lebanon), and 12,100 PRS (40 percent of the Palestinian refugees from Syria in Lebanon) are estimated to be in the Integrated Food Security phase 3 (IPC Phase 3) [27]. The IPC classification system is a standardized tool used to assess and communicate the severity of FI and malnutrition in a given population [26].

Previous reports by UNICEF on child food poverty in 2023 and the “Lebanon Integrated Micronutrient, Anthropometry, and Child Development Survey 2023 (LIMA)” revealed that children’s inability to access and consume a minimum diverse diet in early childhood leads to severe stunting and wasting, which are the most dangerous forms of undernutrition in early childhood [28,29]. The LIMA data clearly indicated that HFI adversely impacts household members’ diets and contributes to the development of child undernutrition and specific micronutrient deficiencies in children, as the prevalence of stunting in children under 5 years was 14% [29]. Additionally, LIMA data showcased that HFI reached 85% among Syrian households, 54% among Lebanese households, and 41% among Palestinian households. 

Building on the insights outlined above, this study delves deeper globally into the interconnected dynamics of household food insecurity, maternal depression, child malnutrition, and differential coping mechanisms adopted by mothers, as it sheds light on these challenges faced in many vulnerable communities, specifically in low- and middle-income countries. These lessons from Lebanon can aid in developing strategies to address similar issues in other regions, contributing to the efforts to improve mental health, promote child well-being, and reduce hunger. Understanding these dynamics highlights how economic instability and hardships affect maternal and child health, offering valuable insights to design effective global interventions.

Given that food insecurity causes and consequences, especially on mental health, are likely context-specific, and because of the scarcity of studies in the Arab region in general and in Lebanon in particular, this study relies on quantitative and qualitative approaches to better investigate the relationship between FI, child malnutrition, and maternal depression, in addition to coping mechanisms used to bolster FI. This study has three aims:

(1) investigate the relationship between HFI and maternal mental health on the one hand, particularly depression;
(2) investigate the relationship between HFI and child malnutrition on the other;
(3) examine the nature of coping mechanisms mothers or caregivers employ in food insecure communities.

## 2. Methodology

### 2.1. Study Design and Participant Recruitment

**Figure 1:**
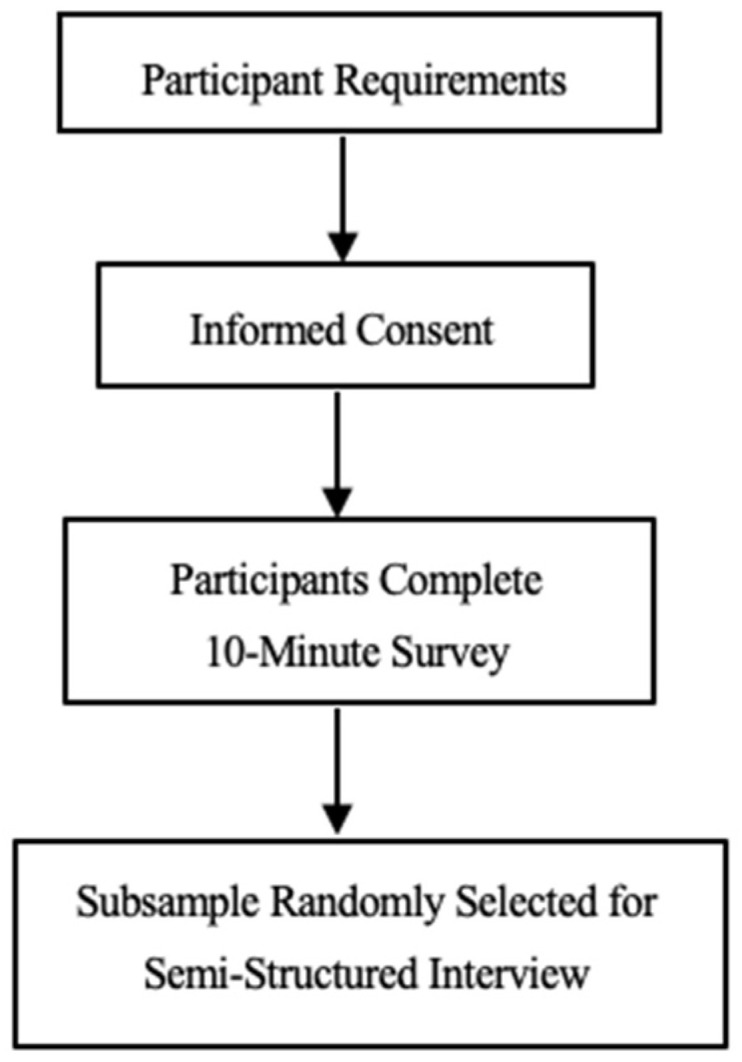
Methodology flowchart.

This is a cross-sectional observational study. Participants were recruited among beneficiaries of a primary health care center (PHCC) in Beirut, the Makhzoumi Foundation, and via recruitment through door-to-door visits in susceptible neighborhoods in Beirut, Mount Lebanon, and South Lebanon. The snowballing technique was adopted among relatives and neighbors. The participants included Lebanese and non-Lebanese mothers of children aged between 6 months and 5 years. Exclusion criteria were mothers under the age of 18 or mothers with children outside the age bracket. Mothers who met the inclusion criteria and agreed to participate in the present study were asked to provide a written informed consent and then asked to complete the survey. They were also informed that they may be contacted later for an interview (cf. Figure 1).

### 2.2. Ethical Considerations

The study protocol and the study tools were approved by the Institutional Board of Review at the Lebanese American University. All participating mothers provided informed consent prior to their participation in the survey and the interview. IRB number LAU.SAS.LM1.4/Nov/2021.

### 2.3. Study Participants: Sample Size and Recruitment

The sample size was calculated based on the assumption that the correlation between the independent and dependent variables differs from zero [30]. An expected correlation coefficient of r = 0.20, a statistical power level of 0.8, and a probability level of 0.05 were specified, resulting in a total sample size of 194. An additional 15% was added to the sample size to account for incomplete data, leaving an intended final sample size of 223 participants.

The subsample size was calculated based on the sample power and showed that in order to collect statistically significant data, 43 participating mothers should be observed. Randomization of the subsample included every third participant for the semi-structured interviews, to make up a total of 57 possible participants, accounting for withdrawals or inability to reach the participant (missing phone number). However, due to the complex contextual situation previously mentioned in the country, including lockdowns and roadblocks, many women were practically unreachable. The final subsample size included 24 women who were given the semi-structured in-person interviews.

### 2.4. Variables Assessment

All participating mothers completed a 10 min survey about their background information, household food insecurity assessment (HFIAS), patient health questionnaire (PHQ-9), and child-related information. A subsample was randomly selected and invited to the PHCC to participate in a semi-structured interview to investigate coping mechanisms.

To limit inter-observer bias, trained research assistants administered the surveys and collected the background data.

1. Sociodemographic and Background Information:

Participants were asked about their sociodemographic (nationality, area of residence, age, child sex, and number of children) and educational statuses. Background information about marital, housing, and employment statuses, sources of income, average monthly salary, and availability of food/money aid was also collected.

2. Household Food Insecurity assessment (HFIAS)

The HFIAS tool was used to screen and assess for the degree of HFI. HFIAS was developed from a short questionnaire that highlights households’ behavioral and psychological manifestations of insecure food access. HFIAS was validated and tested in Arabic to screen for and to assess the degree of HFI across different countries, including Lebanon [31]. It has high reliability and validity in measuring two types of HFI in rural Lebanon: compromised food quality and compromised food quantity [31] The responses to HFIAS determined the degree of severity of insecure food access in the household [32]. The total score ranged from 0 to 27, and the scores were analyzed as categorical variables based on the following cut-off points: (0) food secure, mildly food insecure (1–9), moderately food insecure (10–18), and severely food insecure (19–27). For the current analysis, HFIAS was dichotomized to indicate either the presence or absence of HFI. Those scoring 0–9 were considered as food secure, while those having scores of 10 or higher were categorized as food insecure.

3. Patient Health Questionnaire-9 (PHQ-9):

Depression was assessed using the PHQ-9 [33]. It has been tested extensively for depression screening, it was widely validated as a screening tool in primary care settings in different countries, and its psychometric reliability is established [34]. The validated Arabic version, used in the current study, showed reliable measures of depression screening [35]. It also showed very decent reliability, with a Cronbach alpha coefficient of 0.856 in a recent prevalence study on depression among the Syrian refugee community in Lebanon [36]. The PHQ-9 consists of 9 questions meant to quantify the indicators of depression during the past two weeks on a 4-point Likert scale that ranged from not at all (0) to nearly every day (4). The PHQ-9 score ranged between 0 and 27, with 1–4 indicating minimal depression, 5–9 mild depression, 10–14 moderate depression, 15–19 moderately severe depression, and 20–27 severe depression [33]. For the current analysis, the PHQ-9 variable was used as a dichotomous variable categorizing the participants as either “depressed”, scoring 10–27, or “not depressed”, scoring 1–9 [34].

4. Child Malnutrition Assessment

Mothers of children between 6 months and 5 years were asked to self-report their children’s weight (kg) and height (cm), as it was unsafe to weigh the children or measure their height or middle upper arm circumference due to COVID-19 safety restrictions. If the mother had more than one child in the specified age range, she was asked to report the anthropometric measurements of the child who had the most recent doctor’s visit to minimize recall bias. The recalled measures were plotted on the sex-specific weight to height WHO growth chart (0–5 years) to screen for acute malnutrition. Children were categorized based on the WHO growth chart (kg/cm) as having a normal weight to height ratio, moderate acute malnutrition (MAM) (−2 < z-score < −3), severe acute malnutrition (SAM) (z-score < −3), overweight (1 < z-score < 1.99), or obese (2 < z-score < 2.99). Once again, in our analysis, malnutrition was used as a dichotomous variable categorizing the children as either well-nourished for those who have a normal weight to height ratio or “malnourished” for those identified with SAM, MAM, overweight, or obesity.

### 2.5. Semi-Structured Interview

A qualitative and subjective tool was used to collect information about the coping mechanisms that maternal caregivers have adopted in response to HFI. The semi-structured interview consisted of 7 questions that probed whether mothers had to adopt specific coping mechanisms, the types of behaviors (e.g., dependence on food or money aid), the feeding practices (e.g., breastfeeding) and dietary habits, and the family members who were most affected by HFI, in addition to other reactive habits.

The interview was conducted in Arabic. Mothers were asked for consent to have the interviews recorded, facilitating subsequent thematic analysis.

### 2.6. Statistical Analysis

Statistical analysis was performed using the statistical package IBM SPSS version 29 (SPSS Inc., Chicago, IL, USA), and statistical significance was reported at the conventional level of *p*-value < 0.05. Independent *t*-test or chi-square test for continuous and categorical variables were performed, respectively. Pearson’s correlations were performed to determine the associations between food insecurity, depression, and malnutrition among children at a *p* level of *p* < 0.05. Logistic regression models were used to estimate the odds ratio (OR) and 95% confidence intervals (95% CI) for the association between HFI and depression in a first set and HFI and child malnutrition in a second set. HFI was entered as a categorical variable (food secure vs. food insecure) with food security as a reference, while depression was dichotomized as depressed vs. not depressed (reference variable). The second set of logistic regressions was performed to determine the association between HFI and child malnutrition. The dependent variable, child malnutrition, was dichotomized as well-nourished vs. malnourished (reference variable). To determine the confounders used in the logistic regression, bivariate analysis was performed, and all variables having a *p*-value < 0.2 were included. Accordingly, each of the following variables was entered in the models. When determining depression risk, the variables and reference categories included in the model were place of residence (Beirut), nationality (non-Lebanese), living with partner (yes), husband’s work (employed), educational level (educated), nutritional status (malnourished), HFI (food insecure), number of children < 18 years of age (3 or more), and number of children < 5 years of age (3 or more). When determining the factors affecting the nutritional status, the following variables were included: (HFI) (food insecure), depression (depressed), number of children < 18 years of age (3 or more), number of children < 5 years of age (3 or more), educational level (educated), child sex (male), age, and weight of child in Kgs.

The responses of the semi-structured interview were analyzed qualitatively as themes, clustered from the recorded interviews with the participating subsample.

## 3. Results

### 3.1. Sociodemographic Characteristics

Two hundred and nineteen participants, of which 62 were Lebanese nationals and 157 were non-Lebanese, were included. The non-Lebanese nationalities included Syrians, Palestinians, Sudanese, Bangladeshi, and Egyptians. Moreover, Lebanese mothers were significantly older, had higher education level, were more employed with a personal source of income, and had a lower number of children compared to the non-Lebanese mothers. Additionally, all Lebanese mothers had only 2 children under the age of 5 (n = 0 for ≥3 children), while 8% of non-Lebanese participants had ≥ 3 children under the age of 5 (*p* = 0.021). The sociodemographic characteristics of all participants are presented in Table 1.

The monthly income was reported in both currencies, LBP and USD, due to the severe fluctuation of the exchange rates and the ongoing economic crisis. However, due to the sensitivity of the topic, not all participants answered questions related to monthly income, resulting in non-homogenous variables.

### 3.2. Household Food Insecurity

The mean score of HFI for the whole sample was 12.85 ± 6.56 (Table 1). A total of 70% had moderate or severe HFI, while 30% did not experience HFI. When stratified by nationality, less than half of the Lebanese women (45%, n = 28) were food insecure, while the majority of the non-Lebanese women were food insecure (80%, n = 126, *p* < 0.001).

Additionally, half the non-Lebanese women (58%, n = 91) received food aid, compared to only 26% of the Lebanese (n = 16, *p* < 0.001). The main source of the food aid, for almost half those asked, had to do with cash, food vouchers, or food boxes.

### 3.3. Maternal Depression

Around two thirds of participants (66%, n = 145) had moderate or severe depression, with an average PHQ-9 score of 12.68 ± 6.03. No significant difference was observed based on nationality (Table 1).

### 3.4. Children’s Nutritional Status

Children had a mean age of 2.5 years (mean age = 32.45 months, with a range between 6 months and 5 years) with a mean weight of 13.01 ± 3.92 (Kg) and a mean height of 83.23 ± 14.30 (cm). Lebanese children weighed significantly more (*p* < 0.001) and were significantly taller than non-Lebanese children (*p* = 0.006).

Overall, around two thirds of the children were found to be malnourished (65%, n = 100), experiencing any form of malnutrition: MAM, SAM, obese, or overweight (Table 1).

### 3.5. Association Between Nutritional Status, Household Food Insecurity, and Depression Levels Among Participants

1. Household Food Insecurity and Children’s Nutritional Status

The correlation between the children’s nutritional status and HFI is evaluated in a sample of n = 153, as we had missing data for reported height and weight (Table 2). Interestingly, among those who were food insecure, a little less than half of participants (42%) were reported to have children with acute malnutrition, while 58% were well-nourished (*p*-value = 0.008).

2. Household Food Insecurity and Depression

Regarding HFI and maternal depression, a significant correlation between both variables (*p*-value = 0.001) was observed (Table 2). Among those who were food insecure, the majority (77%) was diagnosed as depressed by the PHQ-9, while among those who were food secure, the majority (60%) were classified as not depressed.

### 3.6. Logistic Regression

1. Depression

In Table 3, results show that compared to those who are food secure, those who were food insecure had an increased risk of depression (OR = 3.162, 95% CI 1.364–7.332, *p* = 0.007). Having 3 or more children aged 5 years and below was also found to decrease the risk of depression (OR = 0.057, 95% CI 0.009–0.367, *p* = 0.003), and having 3 or more children aged 18 years and below was found to increase the risk of depression (OR = 2.485, 95%CI 0.414–3.007, *p* = 0.047).

2. Nutritional Status

No significant association was observed between nutritional status and any of the predictor variables (Table 3).

### 3.7. Qualitative Coping Mechanisms Among Food Insecure Mothers

Mothers reported several coping mechanisms to deal with HFI. These were categorized into themes, including:

1. Self-sacrificial behavior: reducing the portion of their meals, skipping meals, eating last after their children have eaten, eating foods that they do not like, showing patience and endurance for the sake of the children.
2. Limited quantity and quality of the food: setting a specific number of meals a day for the family members and cooking limited types of meals, such as pasta, potatoes, lentils, rice, and legumes, eating the same food more than once a day or over days, adding water and preparing infant milk in a diluted form so that the formula milk lasts longer.
3. Financial/economic restraint: borrowing money to buy food or borrowing food from the supermarket, receiving food from neighbors or relatives when possible, skipping house rent to save money for food, limiting other non-food spending such as clothes, outings, and takeout foods.
4. Distractions from eating: playing games or chatting with the children to forget about food, postponing mealtimes after play time.
5. Negative emotions: feeling of guilt and remorse toward their children, feeling that they are not providing enough for their children, and feeling angry/stressed all the time.
6. Infant and young child feeding (IYCF) misconceptions and practices: believing that breastfeeding of the infants is very nutritious and important to the child’s immunity development, but that its quality is compromised in the context of HFI, as the mother is often malnourished, perceiving breastfeeding as a health burden on the mother that compromises her nutritional status and that breastmilk alone is not a sufficient source of nutrition and cannot satisfy the child’s hunger, introducing milk formula at a young age to supplement breastfeeding or to fully substitute it.
7. Social isolation: isolating children from neighbors or relatives who are more food secure to avoid comparison and only allowing the children to play with people of the same socio-economic status.
8. Paternal buffering mechanisms: fathers spending the whole day at work and only getting to eat twice (in the morning and at night) to spare food for the family members.

## 4. Discussion

Existing literature demonstrates food insecurity to be an important social determinant of mental health [37]; however, less is known about how mothers manage their psychological state in the context of FI when they live in a complex environment with a multilayered crisis context, such as in Lebanon. To the best of our knowledge, this is the first study documenting such variables taken together in unprecedented accumulating challenges in dire socio-economic conditions. In addition to quantitative measurements, this study analyzes in-depth coping strategies used by mothers to dampen the HFI, highlighting various behavioral and cognitive strategies but mostly reflecting gender-bias distribution of food within households.

This study first and foremost shows alarming HFI levels, with only 30% of households being food secure. Segmentation per nationality, furthermore, showed that HFI was highest (80%) among non-Lebanese nationalities. These HFI levels are higher than those reported in recent literature. They go in line with the documented steady increase trend in FI over the years. According to Action Against Hunger, in 2020, 78% of 1.5 million Syrian refugees were suffering from food insecurity [38]. A later country brief by the World Food Program reported that the level of HFI among the Lebanese will increase to 33% of Syrian refugees in 2024.

It is profound to mention the most recent global report on food crisis by the World Food Program, which indicates the rising global food insecurity and how it is impacting mental health and child nutrition [39]. The numbers of people facing these challenges have been increasing every year since 2019. The global food crisis affects both children and women, as 36.4 million children under five years of age suffered from acute malnutrition in 2023 [39]. This does not only affect the nutritional aspect; Food insecurity has significant mental health repercussions, as chronic hunger can lead to anxiety, depression, and impaired cognitive development [39].

### 4.1. HFI and Maternal Depression

The results of this study found a strong positive correlation between HFI and maternal depression; in fact, compared to those who are food secure, those who are food insecure had a higher prevalence of depression in our sample. Consistent with our results, a systematic review and meta-analysis showed an increased risk of depression among maternal caregivers in food insecure households [40]. While FI was originally reported as a predictor of distress in mothers [12], the relationship between food insecurity and maternal depression is further complicated by additional factors and a bidirectional influence [41]. It could be that food-insecure mothers live in constant stress, depleting altogether their physical and cognitive resources. Increased exposure to stress induces heightened cortisol secretion, and inflammation markers could in turn inflate depression symptoms based on the cytokine theory of depression and the lack of basic building blocks of brain neurotransmitters such as serotonin [42]. These nutritional-deficiency-linked depressive symptoms would also be worsened by hypothalamic-pituitary-adrenocortical axis dysfunction due to stress during pregnancy, which increases risks of recurrent depression [43]. Within this framework, women with low educational and income levels were shown to be subjected to higher violence exposure, further increasing the risk of psychological distress, including depression and maladaptive eating behavior and coping mechanisms. These negative coping mechanisms in turn increase the risk of eating disorders (cf. section on coping mechanisms) [44]. As for the number of children, our study showed that mothers with three or more children aged 5 and below had a decreased risk of depression, whereas those with three or more children aged 18 and below had an increased risk of depression compared to mothers with two or fewer children. This finding should be interpreted with caution due to the small sample size of mothers with three or more children in both age groups. However, our results can be understood in the context that mothers with children aged 5 and below may benefit from stronger family connections often available during early childhood, potentially reducing the risk of depression. In contrast, mothers with children aged 18 and below face more complex demands, which could increase their risk of depression.

### 4.2. HFI and Child Malnutrition

In this study, 65% of the sample population had malnourished children. Although 58% of these malnourished children had HFI, there was no correlation between the nutritional status of the child and HFI. This is consistent with a cross-sectional study that found no association between the anthropometrics of children and HFI in 1204 Lebanese households [2]. Conversely, a systematic review and meta-analysis of 21 studies from 12 different countries showed that HFI increased the risk of acute and chronic malnutrition [45]. Another recent meta-analysis revealed that 80% (n = 7) of the studies found a positive correlation between HFI and stunting [46]. The lack of association in our study could be due to several factors. First, both measures, the HFI and nutritional status, are self-reported, leading to recall and social desirability biases, in turn leading to under or inaccurate reporting. Furthermore, since the children’s anthropometric measures were based on the latest doctor’s visit, they might not reflect the recent height or weight. Second, the HFIAS tool assesses FI during the previous four weeks. Hence, the time frame might not be long enough for malnutrition to manifest clinically, or the mothers are still coping, and the effects of FI have not progressed yet. Lastly, as our results showed, the mothers will resort to several coping mechanisms to alleviate FI for their children, particularly resorting to self-sacrificial behaviors such as decreasing the quantities they usually eat so they can properly feed their children. Future studies examining HFI and malnutrition in children should also study the mother’s nutritional status to determine if HFI would have more detrimental effects on the caregivers than on the children.

### 4.3. Mothers’ Coping Mechanisms in FI Communities

Finally, the thematic analysis of the semi-structured interviews provided important insight on maternal coping mechanisms. Women claimed feeling guilt and remorse toward their children and felt that they were failing as mothers. These negative emotions might be mediators toward the association between HFI and depression in mothers [45], but further evidence is needed to test the latter. The 8-themed coping mechanisms that were identified in the scope of this study echo previous findings. A surveillance program targeting more than 4000 households in Yemen had also reported borrowing food to survive, changing types and quality of food, and decreasing the number of meals per day [14]. Financial restraint and economic adaptation by borrowing money or food items from supermarkets, receiving food from neighbors/relatives when possible, and limiting non-food spending such as clothes were all reported in this study. These strategies were previously mentioned [16], as the main coping strategies used by households were reliance on less expensive foods and purchasing food on credit. Strategies that involved financial compromise such as selling or mortgaging assets [47] or sending kids to live in food secure neighboring homes [13] were however not reported in this study.

Interestingly, although maternal self-sacrificial behaviors were recalled both in the literature and in this study, paternal buffering was also reported, with many women claiming that their husbands were the ones mostly affected by food insecurity, as heads of the households who had to prioritize providing for the children and wives over themselves. This coping mechanism contradicts the results of a cross-sectional mixed methods comparative study reported by [17], where maternal buffering and gender power affected intra-household food distribution to the benefit of males. As such, more contextual investigations are needed to clarify such discrepancies, taking culture, religion, employment status, and socio-economic aid into consideration.

It is noteworthy that breastfeeding had a negative connotation in the context of HFI, where it had been described as a burden on mothers and a feeding practice that had poor benefits for the child; and on top of that, it happened at the expense of the mother’ health, well-being, and nutritional status. Such attitudes were not previously reported in studies that had looked at coping mechanisms [48,49,50,51]. The food insecurity created stress, frustration, and despair among women who felt that they could not provide the basic needs (provision of adequate nutrition) to their children and therefore adopted some practical mechanisms to cope. These simple practical techniques varied from distracting children from food through play to socially isolating them, borrowing money, or relying on the absence of fathers so more food is available during the day. Although these mechanisms help alleviate the FI and attend at best to the scarcity of food within the household, they nonetheless do not spare the mental health of mothers, as food insecurity weighs them down and makes them feel sad, helpless, and unable to fulfill their role as “good mothers” [52].

### 4.4. Strengths and Limitations

First, it must be noted that although the data were collected from vulnerable communities residing in different areas inside and outside of Beirut, HFI was screened for using HFIAS. The tool used was also validated in its Arabic version and tested in the Lebanese population. Similarly, the depression screening tool had been extensively used in community setting studies among in- and outpatient clinical settings owing to its translation to several languages, including Arabic, and endorsement for use across various communities [33]. The second strength was the standardization of data collection through training of observers on the survey administration and limiting inter-observer bias. In addition, including two subsets of seemingly homogenous nationality-based populations that are in fact different in quantitative and qualitative reports enhances our observations and allows for future tailored interventional strategies. Lastly, qualitative analysis remains the most innovative approach of the study, investigating the reliance on different practical coping mechanisms by mothers. Taken together with assessment of children’s malnourishment, these data provide invaluable insight to inform government officials and policy makers concerned with food security and child and maternal health.

This study, however, had some limitations that warrant taking the results with care and generalizing cautiously. This study focused only on depression as one type of mental distress, while FI might be conducive to other comorbid psychiatric or psychological illnesses correlated with HFI, such as anxiety and other types of emotions or eating disorders. Also, the data were collected from mothers conveniently and not representatively, which may have affected the generalizability of the results. Convenience and snowballing were the methods of use for data collection due to the challenges and impracticality of transportation in Lebanon at the time. Childhood malnutrition was similarly constrained to subjective evaluations. Nonetheless, these results offer important understanding of FI in an extremely unstable socio-economic and health situation.

## 5. Practical Implications

In conclusion, HFI among Lebanese and non-Lebanese residents is prevalent, with serious repercussions on the mental well-being of mothers and children. This study was able to highlight the positive correlation of HFI with maternal depression, which led to the adoption of practical coping mechanisms in a search to alleviate the burden of child malnutrition. From a practical perspective, our results highlight the need for interventions addressing food insecurity that yield immediate benefits for the mothers and children. It is crucial for such interventions to address the root of the problem, which is primarily financial in the context of Lebanon, and therefore to try to promote communal and local job opportunities to ensure some income for women. This can be done through social enterprise, local community kitchens, and other craftmanship jobs. One very successful example is “Yami”, a women-led initiative in Baalbeck, Lebanon, that emerged in 2020 amidst Lebanon’s economic crisis. Over 100 vulnerable women are involved, producing traditional food (mouneh) and enhancing local food security. Through providing training opportunities on food processing, quality control, and entrepreneurship, Yami empowers women economically, fostering independence and self-sufficiency. Acting as a market for local agricultural produce, it promotes food security and the Mediterranean diet, contributing to community well-being. Yami showcases the transformative power of empowering women in fostering individual and community success. Other implications may follow an up-down approach at the governmental/policy level. Implementing cash transfer programs that target poverty more broadly, as opposed to explicitly food-based distribution initiatives, may prove especially efficacious. Whether it is a policy of cash transfer or providing the women with local employment opportunities, placing these financial resources directly in the hands of women will empower them to allocate funds according to their needs. It is also important that community work focuses on developing proper public health and nutrition education interventions to help suppress the repercussions of food insecurity on infant and young child feeding practices, including breastfeeding, and the physical and mental wellbeing of household members.

## 6. Conclusions

This article is a revised and expanded version of a poster presentation entitled Food Insecurity and Coping Mechanisms: Impact on Maternal Mental Health and Child Malnutrition, which was presented at 45th ESPEN Congress on Clinical Nutrition & Metabolism 2023, Lyon, France [53].

The latest 2024 IPC data [27], which covered the post-data-collection period, clearly indicate that food insecurity in Lebanon is on the rise. Recognizing the responsibilities that women bear in sustaining households and providing for children, particularly in contexts like Lebanon, and acknowledging the increasing body of evidence linking FI with adverse mental well-being outcomes and using coping mechanisms, this study emphasizes the need for ongoing qualitative research on food insecurity as a social determinant of mental health. Including fathers in future studies must also be a priority.

## Figures and Tables

**Table 1:** Characteristics of the Participants as a Whole and Stratified according to Nationality.

Variable	Whole Sample	Lebanese	Non-Lebanese	t (2-Tailed Sig)
**Nationality**				
	219 (100%)	62 (28.3%)	157(71.7%)	-
**Age**				
	30.50 ± 6.20	33.16 ± 45.20	29.45 ± 5.90	<0.001 **
**Area of Residence**				
Beirut	115 (52.5%)	26 (42%)	89 (57%)	0.52
Outside Beirut	104 (47.5%)	36 (58%)	68 (43%)	
**Marital Status**				
Married	216 (99%)	61 (98%)	155 (99%)	1.000
Divorced/Separated	3 (1%)	1 (2%)	2 (1%)	
**Living with Partner**				
Yes	210 (96%)	60 (97%)	150 (96%)	0.158
No	8 (4%)	1 (2%)	7 (4%)	
**Educational Level**				
No Formal Education	25 (11%)	0	25 (16%)	<0.001 **
Middle School or Less	110 (50%)	18 (29%)	92 (59%)	
High School graduate, diploma or the equivalent	50 (23%)	24 (39%)	26 (17%)	
Bachelor’s Degree or Above	34 (15%)	20 (32%)	14 (9%)	
**Employment Status**				
Employed	35 (16%)	22 (35%)	13 (8%)	<0.001 **
Unemployed	184 (84%)	40 (65%)	144 (92%)	
**Personal Income**				
Yes	41 (19%)	25 (40%)	16 (10%)	<0.001 **
No	177 (81%)	37 (60%)	140 (89%)	
**Husband/Partner’s Work**				
Employed	188 (85.8%)	55 (89%)	133 (85%)	0.779
Unemployed	24 (11%)	6 (10%)	18 (11%)	
**Child Sex**				
Female	100 (26%)	31 (31%)	69 (69%)	0.418
Male	119 (54%)	31 (26%)	88 (74%)	
**Number of children < 18 years of age**				
2 or Less	110 (50%)	45 (73%)	65 (41%)	<0.001 **
3 or More	109 (50%)	17 (27%)	92 (59%)	
**Number of children < 5 years of age**				
2 or Less	207 (94%)	62 (100%)	145 (92%)	0.021 *
3 or More	12 (6%)	0	12 (8%)	
**Age in months of children < 5 years**	32.45 ± 15.99	34.19 ± 16.09	31.77 ± 16.01	0.317
**Food/Money Aid**				
Yes	107 (49%)	16 (26%)	91 (58%)	<0.001 **
No	112 (51%)	46 (72%)	66 (42%)	
**Household Food Insecurity (yes, n, %)**				
Mild	54 (25%)	26 (42%)	28 (18%)	<0.001 **
Moderate	110 (50%)	25 (40%)	85 (54%)	
None	11 (5%)	8 (13%)	3 (2%)	
Severe	44 (20%)	3 (5%)	41 (26%)	
Yes *	154 (70%)	28 (45%)	126 (80%)	<0.001 **
No	65 (30%)	34 (55%)	31 (20%)	
**HFIAS Score (n = 219)**	12.85 ± 6.56	8.92 ± 6.43	14.40 ± 5.97	<0.001 **
**Children Nutritional Status**				
**Well-nourished**	53 (35%)	19 (38%)	34 (33%)	0.589
**Malnourished**	100 (65%)	31 (62%)	69 (67%)	
**Weight in kgs (n = 191)**	13.01 ± 3.92	14.52 ± 4.04	12.29 ± 3.68	<0.001 **
**Height in cm (n = 158)**	83.23 ± 14.29	87.69 ± 14.75	81.04 ±13.68	0.006 *
**Depression**				
**Depressed**	145 (66%)	36 (58%)	109 (69%)	0.116
**Not depressed**	74 (34%)	26 (42%)	48 (31%)	
**PHQ-9 Score (n = 219)**	12.68 ± 6.03	11.85 ± 6.21	13.01 ± 5.96	0.205

(1) Abbreviations: yes * in household food insecurity is a combination of moderate and severe cases; HFIAS score: household food insecurity assessment; PHQ-9 score: patient health questionnaire. (2) symbols: * Statistically significant (*p* < 0.05), ** Strongly statistically significant (*p* < 0.05). (3) ±: Standard deviation.

**Table 2:** Association between Nutritional Status, Household Food Insecurity, and Depression Levels among Participants.

		Household Food Insecurity		Pearson Chi-Square
		Yes	No	
**Children’s Nutritional Status (n = 153)**	**Well-Nourished**	43 (42%)	10 (20%)	0.008 **
	**Malnourished**	60 (58%)	40 (80%)	
**Depression (n = 219)**	**Depressed**	119 (77%)	26 (40%)	0.001 **
	**Not depressed**	35 (23%)	39 (60%)	

symbols: ** statistically significant (*p* < 0.05).

**Table 3:** Determinants of Maternal Depression and Children’s Nutritional Status.

Variables	Depression (n = 219)			Nutritional Status (n = 147)		
	OR	95% CI	*p*-Value	OR	95% CI	*p*-Value
Place of residence Beirut (1) Outside Beirut (0)	0.566	0.243–1.318	0.848	-	-	
Nationality Lebanese (1) Non-Lebanese (0)	1.116	0.414–3.007	0.83	-	-	
Living with partner Yes (1) No (0)	1.131	0.042–30.488	0.942	-	-	
Husband’s Work Un-employed (1) Employed (0)	2.091	0.547–8.001	0.281	-	-	
Educational Level Educated (1) No formal education (0)	0.894	0.347–2.301	0.816	0.546	0.166–1.801	0.320
Nutritional Status Malnourished (1) Well-nourished (0)	0.934	0.384–2.268	0.88	-	-	
**Household Food Insecurity** **Food Insecure (1)** **Food Secure (0)**	**3.162**	**1.364–7.332**	0.007 *	0.568	0.215–1.503	0.260
**Number of children < 18 years of age** **3 or more (1)** **2 or less (0)**	**2.485**	**0.414–3.007**	0.047 *	2.349	0.898–6.140	0.080
**Number of children < 5 years of age** **3 or more (1)** **2 or less (0)**	**0.057**	**0.009–0.367**	0.003 *	0.182	0.018–1.896	0.150
Depression Depressed (1) Non-depressed (0)	-	-	-	0.550	0.208–1.452	0.230
Child Sex Male (1) Female (0)	-	-	-	0.438	0.191–1.006	0.052
Age	-	-	-	0.990	0.926–1.059	0.780
Weight of child in Kgs	-	-	-	1.068	0.953–1.198	0.260

Symbols: * Statistically significant (*p* < 0.05).

## Data Availability

The original contributions presented in this study are included in the article. Further inquiries can be directed to the corresponding author.

## Abbreviations

FI	Food insecurity
HFI	Household food insecurity
PHCC	Primary health care center
HFIAS	Household food insecurity assessment
PHQ-9	Patient health questionnaire
MAM	Moderate acute malnutrition
SAM	Severe acute malnutrition
